# Design and Preclinical Evaluation of Nicotine–Stearic Acid Conjugate-Loaded Solid Lipid Nanoparticles for Transdermal Delivery: A Technical Note

**DOI:** 10.3390/pharmaceutics15041043

**Published:** 2023-03-23

**Authors:** Jwala Renukuntla, Samuel Peterson-Sockwell, Bradley A. Clark, Nipunika H. Godage, Emanuela Gionfriddo, Pradeep Kumar Bolla, Sai H. S. Boddu

**Affiliations:** 1 School of Pharmacy, The University of Texas at El Paso, 1101 N Campbell St., El Paso, TX 79902, USA; 2 Department of Basic Pharmaceutical Sciences, Fred Wilson School of Pharmacy, High Point University, High Point, NC 27240, USA; 3 Department of Chemistry and Biochemistry, School of Green Chemistry and Engineering, College of Natural Sciences and Mathematics, University of Toledo, Toledo, OH 43606, USA; 4 College of Pharmacy and Health Sciences, Ajman University, Ajman P.O. Box 346, United Arab Emirates; 5 Centre of Medical and Bio-Allied Health Sciences Research, Ajman University, Ajman P.O. Box 346, United Arab Emirates

**Keywords:** nicotine, stearic acid, solid lipid nanoparticles, gel, transdermal permeation, smoking cessation

## Abstract

This study aimed to develop and evaluate nicotine--stearic acid conjugate-loaded solid lipid nanoparticles (NSA-SLNs) for transdermal delivery in nicotine replacement therapy (NRT). Nicotine conjugation to stearic acid prior to SLN formulation greatly increased drug loading. SLNs loaded with a nicotine–stearic acid conjugate were characterized for size, polydispersity index (PDI), zeta potential (ZP), entrapment efficiency, and morphology. Pilot in vivo testing was carried out in New Zealand Albino rabbits. The size, PDI, and ZP of nicotine–stearic acid conjugate-loaded SLNs were 113.5 ± 0.91 nm, 0.211 ± 0.01, and −48.1 ± 5.75 mV, respectively. The entrapment efficiency of nicotine–stearic acid conjugate in SLNs was 46.45 ± 1.53%. TEM images revealed that optimized nicotine–stearic acid conjugate-loaded SLNs were uniform and roughly spherical in shape. Nicotine–stearic acid conjugate-loaded SLNs showed enhanced and sustained drug levels for up to 96 h in rabbits when compared with the control nicotine formulation in 2% HPMC gel. To conclude, the reported NSA-SLNs could be further explored as an alternative for treating smoking cessation.

## 1. Introduction

Tobacco consumption can lead to several fatal lung diseases including chronic obstructive pulmonary disease and cancer [1,2]. Each year in the United States alone, nearly half a million people die from tobacco use [3]. Tobacco contains the highly addictive substance nicotine, a toxic alkaloid that stimulates neural and cardiovascular systems at lower doses and depresses them at higher doses [4]. Smoking-related illnesses cost approximately $170 billion each year [3]. While smoking tobacco use in the US has recently declined, electronic nicotine delivery system (ENDS or e-cigarette) usage has become more popular [5,6,7]. Of particular concern, the Centers for Disease Control and Prevention (CDC) reports that e-cigarettes have become the most used nicotine product among middle and high school students [8]. It is well-established that smokers who utilize nicotine replacement therapies (NRTs) to quit are more likely to be successful than those who do not [5], and an increased rate of abstinence among smokers has been observed with NRTs [2]. Therefore, NRT has been recommended as a first-line treatment for smoking cessation [5,6]. NRTs deliver nicotine to the systemic circulation and are available in a variety of delivery systems [5]. Therapeutic delivery systems marketed for NRT are proportioned as follows: 54% are chewing gum-based products; 19% are lozenges; 23% are transdermal patches; and the remaining 4% are inhalers, nasal sprays, mouth sprays, and orodispersible film strips [9,10]. NRTs are available in varying strengths: the gum in 2 and 4 mg; the lozenges in 1, 1.5, 2, and 4 mg; inhalers in 10 and 15 mg; and nasal sprays in 0.5 of 1 mg per actuation [11]. Oral nicotine delivery is associated with side effects such as hiccups, sore mouth, throat irritation, cough, gastrointestinal discomfort, and nausea. These effects manifest partly because nicotine, whether by intention of or as a byproduct of the design, goes through the gastrointestinal tract and subsequent first-pass metabolism. This necessitates increased oral nicotine doses in order to overcome the resulting decrease in bioavailability, thus resulting in unwanted side effects [12,13].

While nicotine nasal spray relieves withdrawal symptoms very quickly, at least eight doses (16 sprays) each day may be needed to control nicotine cravings. In addition, nasal irritation, runny nose, watery eyes, sneezing, nasal burning, throat irritation, and coughing are some of the most commonly reported side effects [14]. Nicotine transdermal patches avoid many of these side effects by bypassing first-pass metabolism and delivering nicotine through the skin directly into the systemic circulation [15]. Generally, these transdermal patches are available in strengths of 7 mg per day, 14 mg per day, and 21 mg per day. Clinical trials conducted for nicotine patches have demonstrated a reduction in withdrawal symptoms and craving for nicotine [16]. In addition, the possibility of long-term abstinence with patches has increased relative to a placebo [16,17,18,19]. Constant levels of nicotine can be achieved via transdermal patches [16], but this mode of administration delivers nicotine dose slowly and fails to replicate the immediate delivery that oral or other formulations produce [11]. Nicotine concentrations resulting from patches are usually half of the concentrations obtained by smoking. The most common side effects reported with patches include skin irritation and sleep disturbances [20]. Patient compliance with the currently marketed nicotine patches is low due to numerous factors such as side effects, costs, visibility, and the misconception that NRT is ineffective [21,22,23]. Furthermore, advances in research and development are needed to address the existing challenges associated with NRT delivery systems.

Recently, researchers have explored several nanoparticle-based formulations of nicotine delivery through nasal and oral routes. Solid lipid nanoparticles (SLNs) and nanostructured lipid carriers (NLCs) have received strong interest and attention in drug delivery design due to their ability to decrease drug toxicity, prevent the burst release of drugs, augment drug stability, solubilize poorly soluble drugs, modulate drug release with flexibility, achieve sustained- and controlled-release profiles, and provide targeted delivery [24]. SLNs and NLCs also improve the penetration of drugs across the skin because of the formulation’s lipid’s interaction with skin lipids and the hydrating occlusive nature of lipids used in the preparation [25]. In addition, the nanosize of SLNs and NLCs facilitates the spread of drugs across the skin surface and thus increases drug penetration through the skin surface [26,27]. In 2018, Ding et al. reported a method optimizing nicotine-loaded SLNs for oral delivery using Kolliwax^®^ S and hydrogenated sunflower oil as lipids [28]. Lipid conjugate of drugs are known to provide improved bioavailability [29]. Lipid-based nanostructures have been widely explored for topical and transdermal delivery.

In this paper, considering these factors, we envision that nicotine-loaded SLNs (NicSLNs) could be an attractive transdermal delivery system for smoking cessation purposes. Nicotine–stearic acid (NSA) conjugate was used in order to increase the entrapment efficiency into SLNs. SLNs were prepared using hot homogenization and ultrasonication method. SLNs were characterized for size, polydispersity index (PDI), zeta potential (ZP), and entrapment efficiency. NSA conjugate-loaded SLNs (NSA-SLNs) were prepared to enhance the entrapment efficiency. Furthermore, NSA-SLNs were tested in vivo following transdermal application in New Zealand Albino rabbits. NSA-SLNs (test) or NSA conjugate (control) were dispersed in 2% of hydroxypropyl methylcellulose (HPMC) gel to facilitate their application on rabbit skin as a gel formulation.

## 2. Materials and Methods

### 2.1. Materials

Compritol 888 ATO was a generous gift from Gattefossé (Saint-Priest Cedex, France). Poloxamer 188 and stearic acid were purchased from Alfa Aesar (Ward Hill, MA, USA). Nicotine was procured from Acros Organics (Fair Lawn, NJ, USA). All other chemicals and solvents were of analytical grade and were obtained from Fisher Chemicals (Fair Lawn, NJ, USA). Hydroxypropyl methylcellulose (HPMC) (Methocel^®^ E4M premium CR (hypromellose USP) (Lot C143851) was purchased from PCCA (Houston, TX, USA). An in-house Milli-Q IQ 7000 water purification system by Merck (Burlington, MA, USA) was used to obtain milli-Q water (resistivity of 18.2 MΩ).

### 2.2. Methods

#### 2.2.1. Preparation of SLNs

Initially, SLNs with different concentrations of poloxamer (0.5% to 2%) and nicotine loading (0.1% to 1%) were evaluated to optimize the formulation. SLNs were prepared using hot homogenization and ultrasonication techniques, with slight modifications. In brief, Compritol 888 ATO (solid lipid, 500 mg) was melted at 72 °C and an accurately weighed quantity of nicotine or NSA conjugate was added to obtain a clear solution at 72 °C. NSA conjugate was synthesized using a previously reported method [28]. Aqueous poloxamer 188 solution, preheated to 72 °C, was added to the molten lipid solution and homogenized at 5000 rpm for 5 min using a Fisher Scientific™ 850 Homogenizer. After homogenization, the emulsion formed was sonicated for 5 min at 30% amplitude using a Fisher Scientific™ Model 505 Sonic Dismembrator and then suddenly cooled to 20 °C using an ice bath. Gel formulations for in vivo rabbit study were prepared by dispersing NSA-SLN or NSA conjugate containing 5 mg/mL of nicotine in a 2% HPMC dispersion prepared by mixing 400 mg of HPMC in 20 mL water. A uniform gel formulation was achieved through continuous stirring.

#### 2.2.2. Preparation of Nicotine–Stearic Acid (NSA) Conjugate

**Figure 1:**
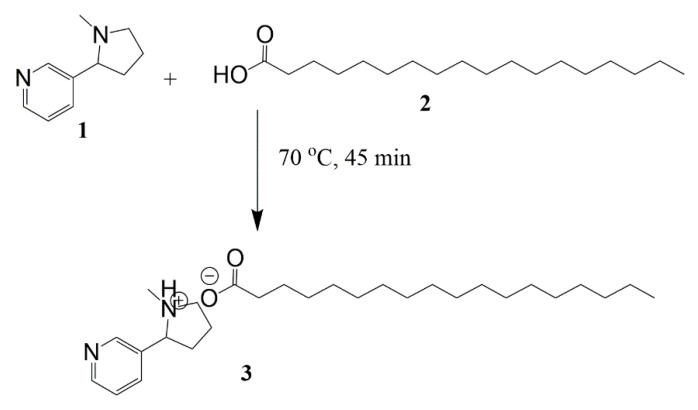
Synthesis of the nicotine–stearic acid conjugate.

An alternative approach for enhancing the entrapment of hydrophilic molecules is to increase the hydrophobicity by conjugating it with an ionic lipid. Recently, Ding et al. increased the lipophilicity of nicotine by forming a lipid–drug conjugate using stearic acid and Kolliwax^®^ S. Their method was adapted and applied to synthesize the NSA conjugate by heating molar ratios of nicotine and stearic acid (1:1.2) at 70 °C for 45 min (Figure 1). The color of liquid mixtures turned dusky red, demonstrating the conjugation of nicotine with stearic acid [28]. The resultant mixture was cooled to room temperature and the NSA conjugate was used for entrapping inside the SLNs.

#### 2.2.3. Particle Size and ZP Analysis by DLS

Particle size and PDI measurements were performed using a Malvern zeta sizer (Zeta sizer NanoZS, Malvern Instruments Ltd., Worcestershire, UK) at 25 °C and at 173° angle to the incident beam by applying the principle of photon correlation spectroscopy (PCS). ZP was measured using electrophoretic mobility housed within the same instrument. SLN dispersions (200 µL) were diluted in 10 mL of milli-Q water before the analysis. All of the measurements were performed on three different samples prepared on the same day.

#### 2.2.4. Percentage Entrapment Efficiency (%EE) and Loading Capacity (%LC)

The percentage entrapment efficiency (%EE) of nicotine loading into SLNs was determined by the ultrafiltration method using centrifugal filter tubes (molecular weight cut-off: 10 kDa) (Amicon Ultra^®^-0.5 mL) [30,31] by placing 0.5 mL of SLN suspension into a centrifugal filter tube and centrifuging the samples at 10,000 rpm for 20 min at 20 °C. The amount of unentrapped nicotine in the aqueous phase (filtrate) was measured by UV–visible spectroscopy (Shimadzu, Kyoto, Japan) at λ_max_ of 261 nm. The following equations were used for calculating the entrapment efficiency and loading capacity:

$$\mathrm{Entrapment}\;\mathrm{efficiency}\;(\%\mathrm{EE})=100\times [\frac{W_{i}-W_{f}}{W_{i}}]$$

*W_i_* is the amount of nicotine initially added and *W_f_* is the mount of free nicotine.

$$\mathrm{Loading}\;\mathrm{capacity}\;(\%\mathrm{LC})=100\times \left[\frac{W_{i}-W_{f}}{W_{lipid}+W_{i}}\right]$$

*W_i_* is the amount of nicotine initially added, *W_f_* is the amount of free nicotine, and *W_lipid_* is the amount of lipid added.

#### 2.2.5. Scanning Transmission Electron Microscopy

Scanning transmission electron microscopy (STEM) (Hitachi HD-2300A) was used to study the surface morphology of the NSA-SLNs. For this, a drop of diluted SLN was placed on the gold grids and any excess dispersion on the gold grid was wicked using filter paper. Then, the grid was placed for drying in a sterile fume hood for two days. Finally, the sample grid was transferred into the STEM and high-resolution images were recorded in secondary electron (SE) image mode at an accelerated voltage of 200 kV.

#### 2.2.6. Differential Scanning Calorimetry (DSC)

Calorimetric analysis for NSA conjugate, lyophilized SLNs, Compritol 888 ATO, Poloxamer 188, and blank SLNs was performed using a DSC822e (Mettler Toledo GmbH, Schwerzenbach, CH, USA) instrument. Each sample was accurately weighed (3–8 mg) in 40 µL aluminum pans and hermetically sealed using a crimping device. Nitrogen was used as a purge gas and was pumped at a flow rate of 20 mL/min. All of the samples were held at 0 °C isotherm for 5 min, then heated at 10 °C/min from 0 °C–100 °C, 100 °C–0 °C, 0 °C–100 °C, and finally 100 °C–0 °C. An empty pan was used as the reference on the opposite side. All of the thermograms were analyzed using Mettler STARe software.

#### 2.2.7. X-ray Diffraction (XRD)

XRD was performed to study the polymorphic changes in the lipids and drugs used in the formulation of SLNs [32]. The analysis was carried out using Rigaku Miniflex X-ray Diffractometer (Rigaku Corporation, Tokyo, Japan). A double-sided adhesive tape was applied over the sample holder and powdered (ground) samples were poured onto the sample holder using a thin spatula. The intensity of the diffracted beam was analyzed in a 2θ range between 10° and 70°. All of the samples were analyzed using JADE software (Livermore, CA, USA).

#### 2.2.8. Physical Stability Studies

Short-term physical stability studies were conducted by storing the SLNs at 4 °C and room temperature (RT) for 30 days. The evaluation of the physical appearance, size, PDI, and ZP of SLNs was performed. These kind of short-term stability studies for 30 days have been reported in the literature for lipid-based drug delivery systems [33].

#### 2.2.9. Testing of Nicotine Formulations in Rabbits

**Figure 2:**
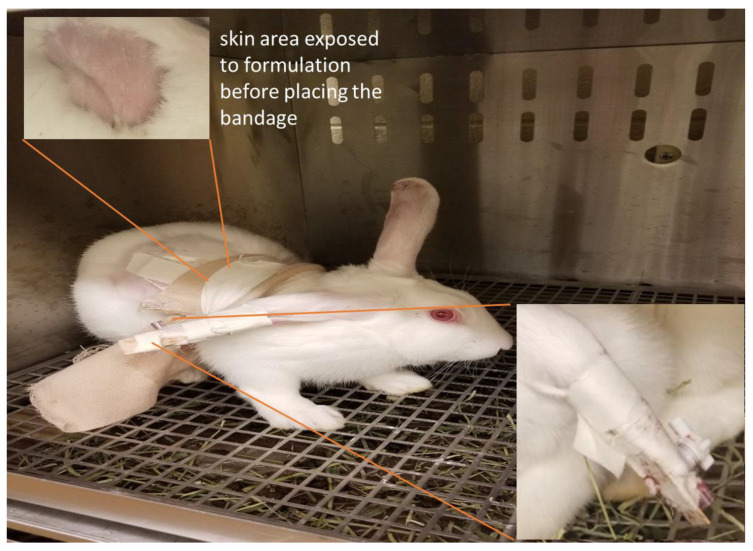
Image of rabbit after the catheter was fixed on the central ear artery with a three-way gate valve and further the assembly was supported by the popsicle stick and tape (in-set).

A pilot study was conducted in rabbits to examine the ability of the transdermal gel to improve the nicotine pharmacokinetic parameters. New Zealand Albino rabbits (weighing 2–4 kg) were procured from Robinson Services Incorporated (Mocksville, NC, USA) and housed in accordance with the Association for Assessment and Accreditation of Laboratory Animal Care International (AAALACI) and U.S. Department of Agriculture (USDA). The animal protocol was reviewed and approved by the Institutional Animal Care and Use Committee (IACUC) at the University of Toledo. The rabbits were randomly assigned to two different groups (seven rabbits/group). The temperature, heart rate, and respiratory rates were measured before the study. The rabbits were shaved on the lateral body side for the topical application of the formulation. About 2 g of control formulation (a dose of 10 mg of nicotine) was applied to each rabbit. One group received the control formulation containing 5 mg/mL of nicotine in 2% of hydroxypropyl methylcellulose (HPMC) gel, while the other group received the test gel (5 mg/mL) containing NSA conjugate loaded in SLNs. The skin area exposed to the formulation was secured with a tape to mimic the patch. The rabbits were administered intramuscular acepromazine (1 mg/kg) as a vasodilator/tranquilizer half an hour prior to blood collection, as needed. The catheter was fixed on the central ear artery with a three-way gate valve (one for drawing blood syringe, one for heparin flux, and one connected to the catheter). The assembly was supported by the popsicle stick and tape (Figure 2). At regular time intervals of 0, 1, 3, 6, 12, 24, 36, 48, and 72 h, approximately 1 mL of blood was placed in heparinized tubes. The gate-valves were flushed with heparin solution after each sampling to prevent clot formation in the catheter. In case of clogging of catheters with clots, sampling was carried out from marginal ear veins. The rabbits were euthanized after completing the study using an IV injection of beuthanasia through the marginal ear vein. The blood samples were centrifuged at 6000 RPM (2000× *g*) for 5 min for the separation of plasma from the blood cells, and the plasma samples were further stored in freezers at −80 °C until extraction. The samples were thawed at room temperature for the extraction of nicotine. The determination of nicotine in rabbit plasma was achieved by the solid phase microextraction–liquid chromatography–tandem mass spectrometry method, as described in our previous publication [34].

#### 2.2.10. Statistical Analysis

Statistical analysis of data was performed using GraphPad Prism (version 5.0) (GraphPad Software, La Jolla, CA, USA). T-test followed by non-parametric Mann–Whitney test and paired *t*-test were applied to analyze the data. A *p*-value of <0.05 was considered statistically significant.

## 3. Results

### 3.1. Optimization of SLN Formulation

#### 3.1.1. Effect of Poloxamer 188 Concentration

Compritol 888 ATO was selected as a solid lipid for SLNs as it is widely used in developing sustained release formulations of highly water-soluble active molecules [35]. Moreover, the entrapment efficiency with Compritol is greater because of the high amounts of mono-, di-, and tri-glycerides in its chemical structure [36]. Poloxamer 188 was chosen as a stabilizer for the formulation due to its high ratio of hydrophilic blocks and low molecular weight [37]. It also has the ability to coat the nanoparticles effectively and impart stealth properties [30]. Initially, the effect of Poloxamer 188 concentration on the size, PDI, and ZP of blank SLNs was studied to optimize the formulation. In our study, we observed that the particle size of SLNs reduced significantly with an increase in the concentration of Poloxamer 188 from 0.5% *w*/*w* to 2% *w*/*w* (*p* < 0.05) (Table 1). The results were in alignment with previous findings where there was a decrease in the particle size of SLNs with an increase in the surfactant concentration [31]. The decrease in the mean particle diameter of SLNs with the increased concentration of Poloxamer 188 was due to the active surface properties [38]. However, there was no significant effect on the size of SLNs at the 1.5% *w*/*w* and 2.0% *w*/*w* concentration (*p* > 0.05). Therefore, Poloxamer 1.5% *w*/*w* was considered as the optimum concentration for the formulation of nicotine-loaded SLNs.

#### 3.1.2. Effect of Nicotine Loading

The effect of nicotine loading was evaluated by measuring the entrapment efficiency of nicotine-loaded SLNs. Different quantities of nicotine (0.1% to 1%) were loaded into an optimized blank SLN formulation. The size, PDI, ZP, and entrapment efficiency of various nicotine-loaded SLNs are provided in Table 2. Although the size, PDI, and ZP values were acceptable (<150 nm) for all of the formulations, entrapment was very low with high standard deviations within each sample set. The results showed that the entrapment efficiency was less than 20% for all of the concentrations. The low entrapment efficiency could be attributed to the hydrophilic nature of nicotine, which limits its entrapment into lipids. The results obtained in our study were consistent with other reports evaluating the entrapment of nicotine into lipid-based drug delivery systems [27].

#### 3.1.3. Preparation of NSA Conjugate and Loading into SLNs

One hundred percent of the input was recovered at the end of the conjugation process in the form of the NSA conjugate. Stearic acid and nicotine freely intermingled and the preparation process gradually redistributed the ions in such a way that the stearic acid interfaced with the nicotine, and they formed a weak but stable affinity and were conjugated. As the mixture cooled and solidified, a more stable conjugated state was formed, which was indicated by a slight color change (first seen in the liquid and then maintained in the solid). The formed NSA conjugate was loaded into SLNs to increase the entrapment efficiency. The formula for optimized NSA-SLNs is presented in Table 3. As expected, the entrapment efficiency of nicotine in SLNs increased significantly to 46.45 ± 1.53%, and the loading capacity increased to 4.64 ± 0.15%. The higher entrapment efficiency of NSA was attributed to the increase in the lipophilicity of nicotine through conjugation with stearic acid. The enhancement of the percent entrapment efficiency was similar to the previous results reported by Ding et al. [28], where the entrapment efficiency was 54% for nicotine-loaded W-SA-LDC-SLNs. The size, PDI, and ZP of the nicotine–stearic acid conjugate-loaded SLNs were 113.5 ± 0.91 nm, 0.211 ± 0.01, and −48.1 ± 5.75 mV, respectively.

### 3.2. Surface Morphology of SLNs

**Figure 3:**
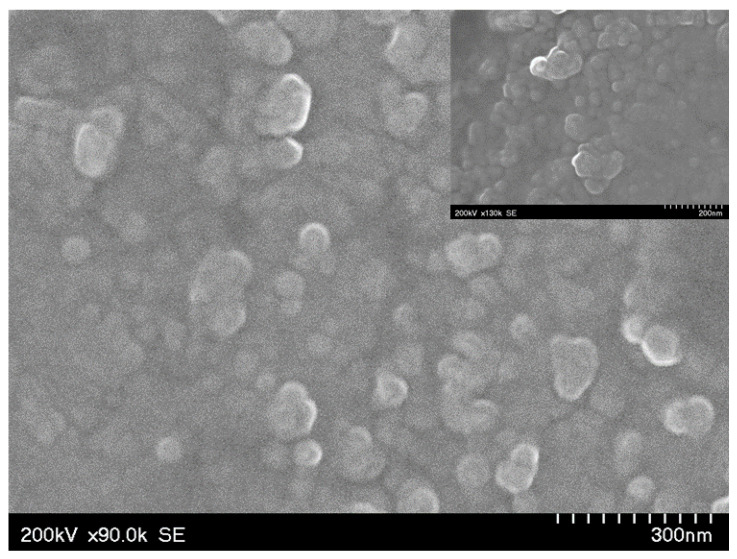
Scanning TEM image (SE mode) of nicotine–stearic acid conjugate-loaded SLNs. A slightly higher resolution image is provided in the inset.

The surface morphology analysis by scanning TEM revealed that the shape of NSA-SLNs was roughly spherical with a uniform size distribution, as shown in Figure 3. The size of the NSA-SLN samples was related to the particle size data obtained from the DLS study.

### 3.3. Differential Scanning Calorimetry (DSC)

**Figure 4:**
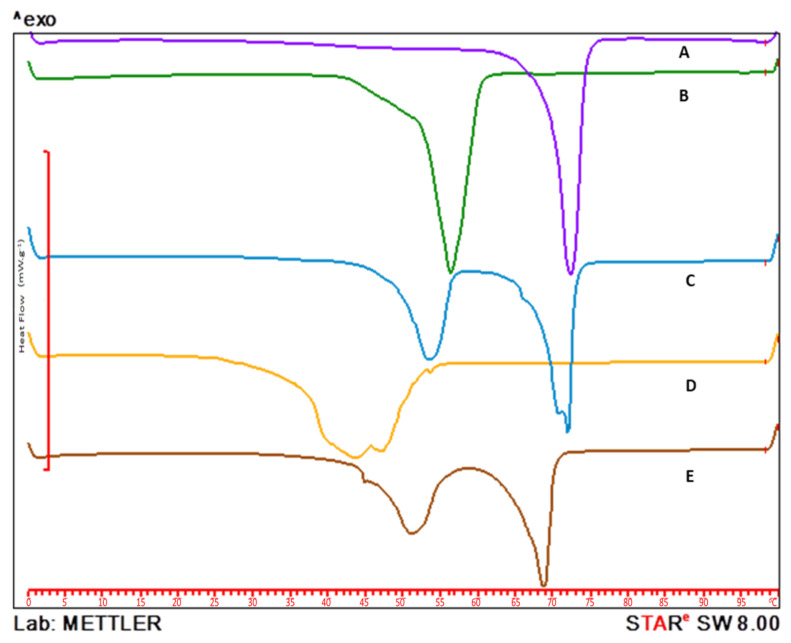
Overlaid DSC thermograms of (A) Compritol 888 ATO, (B) Poloxamer 188, (C) blank SLNs, (D) nicotine–stearic acid conjugate, and (E) lyophilized nicotine–stearic acid conjugate-loaded SLNs.

Overlaid DSC thermograms showing the melting behavior of Compritol 888 ATO, Poloxamer 188, NSA conjugate, and lyophilized blank and NSA-SLNs are provided in Figure 4. A sharp endothermic peak was observed for Compritol 888 ATO and Poloxamer 188 at 70 °C and 52 °C, respectively. A broader peak for the NSA conjugate was observed with a melting point of around 45 °C. The DSC thermogram of the NSA conjugate-loaded SLNs showed the absence of the characteristic peak of NSA, confirming the entrapment of the NSA conjugate into the SLNs. However, there was a slight shift in the peaks corresponding to Compritol 888 ATO and Poloxamer 188 in the NSA-SLN thermogram.

### 3.4. X-ray Diffraction

**Figure 5:**
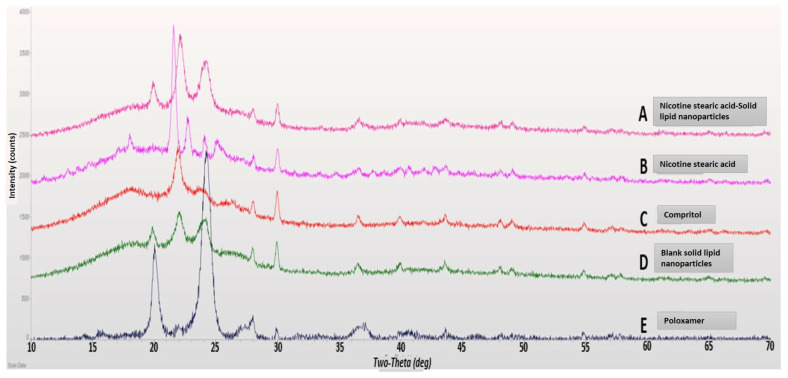
Overlaid XRD pattern: (A) lyophilized nicotine–stearic acid loaded SLNs, (B) nicotine–stearic acid conjugate, (C) Compritol 888 ATO, (D) blank SLNs, and (E) Poloxamer 188.

XRD studies are conducted to study the crystalline nature of drug molecules and can be used to complement DSC results [39,40]. Here, the results showed that the characteristic peak associated with the NSA conjugate was absent in the XRD data of lyophilized NSA-SLNs (Figure 5). This confirmed the non-crystalline nature of NSA-SLNs. Overall, the XRD results complemented the DSC results, confirming the entrapment of the NSA conjugate into the SLNs.

### 3.5. Short Term Physical Stability

The stability study results showed no significant difference in the physical appearance (change in color) or in the particle size of SLNs at day 0 and day 30 (*p* > 0.05) (Table 4). There was a slight increase in the particle size of SLNs during the storage time at both temperatures. For the samples stored at RT, the particle sizes at day 0 and day 30 were 113.5 ± 0.91 nm and 116.0 ± 3.366 nm, respectively. For the samples stored at 4 °C, the particle sizes at day 0 and day 30 were 113.5 ± 0.91 nm and 128.3 ± 10.40 nm, respectively. No significant difference in PDI and ZP values during the storage period also confirmed the stability of SLNs (*p* > 0.05).

### 3.6. Analysis of Rabbit Plasma Samples

**Figure 6:**
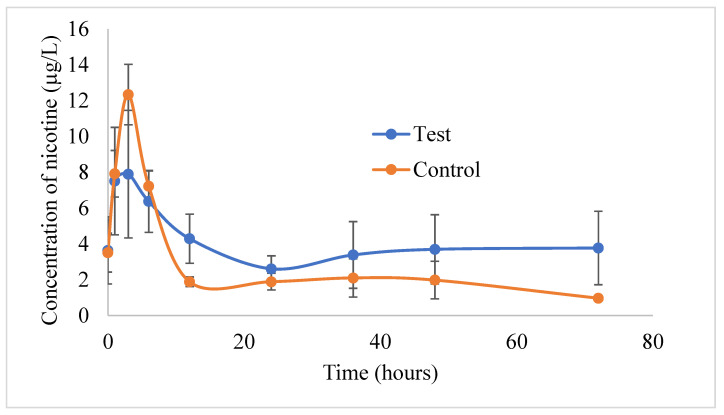
Plasma concentrations of nicotine in rabbits following transdermal application of the control and test gel formulations. Values are represented as mean ± S.E.M, *n* = 4.

Figure 6 shows the nicotine concentrations of nicotine in the rabbit plasma samples from the control and test formulations. PKSolver, a Microsoft Excel program, calculated the pharmacokinetic parameters. A noncompartmental analysis for plasma data following extravascular administration was used to identify parameters such as the AUC_(0–t)_, C_max_, and t_max_ values. The C_max_ values of nicotine from the control and test gel formulations were found to be 12.33 ± 1.68 µg/L and 8.75 ± 3.34 µg/L, respectively. The C_max_ value was achieved after 3 h in both formulations. The test gel containing NSA-SLNs showed enhanced and sustained drug levels for up to 96 h when compared with the control nicotine formulation in 2% HPMC gel. Although the plasma concentration of nicotine from the control gel formulation was found to be slightly higher compared with the test formulation during the initial time points (1, 3, and 6 h), the test formulation showed higher nicotine plasma concentrations in the later time points. This was also evident with the AUC_(0–t)_ values. The AUC_(0–t)_ valued of nicotine from the control and test gel formulations were found to be 188.96 ± 33.83 µg/L*h and 283.73 ± 123.77 µg/L*h, respectively. This clearly indicates that the test gel containing NSA-SLNs showed sustained permeation of nicotine through the rabbit skin. A similar trend was observed in in vitro permeation in the Strat-M^®^ membrane using a PermeGear^®^ ILC-07 automated system (data not presented). At all-time points, the permeation of nicotine from NSA-SLN gel showed a sustained effect compared with pure nicotine gel. We did not expect any toxicity from the gel formulation of NSA-SLNs as the excipients used in the preparation of SLNs such as poloxamer and Compritol 888 ATO are approved for use in topical formulations. Poloxamer 188 is used as gelling agent at a concentration of 15–50% *w*/*w*, while Compritol 888 ATO is used as viscosity-increasing agent in w/o or o/w emulsions at a concentration of 1.0–5.0% *w*/*w* [41].

The literature reveals that nicotine is slowly absorbed from the marketed transdermal patches, with venous nicotine levels peaking around 6 to 10 h post-administration. Moreover, after 24 h of patch administration, the nicotine levels steadily declined from a peak to trough of 25% to 40%, respectively [42]. With the preliminary data obtained from this study, it is possible to assume that NSA-SLNs would be beneficial for maintaining steady levels of nicotine for a prolonged period of time. Yet another possibility would be to design a gel formulation consisting of both free nicotine and NSA-loaded SLNs. From such a system, the initial drug release can be expected from the free drug, while NSA-SLNs can provide an additional drug release in a sustained fashion, thereby improving the overall efficiency of the system.

## 4. Conclusions

The entrapment of drug molecules into SLNs resulted in a sustained release profile. In this study, nicotine–stearic acid conjugate-loaded SLNs were successfully prepared and characterized. The entrapment efficiency of pure nicotine was less than 20%; however, NSA conjugation increased the entrapment efficiency to 46.45 ± 1.53%. The particle size, polydispersity index, and zeta potential of the optimized SLNs were 113.5 ± 0.91 nm, 0.211 ± 0.01, and −48.1 ± 5.75 mV, respectively. NSA-SLNs showed sustained permeation of nicotine through the rabbit skin for up to 96 h. In addition, the higher AUC value of NSA-SLN gel indicates the increased bioavailability of nicotine in rabbits compared with the nicotine gel formulation. Based on the results from this study, it can be concluded that NSA-SLN gel could be considered as a potential treatment option for NRT. However, the findings from this study are preliminary and should be further confirmed with a detailed preclinical study.

## Figures and Tables

**Table 1:** Effect of Poloxamer 188 concentration on the size and PDI of SLNs (*n* = 3).

Poloxamer 188 (% *w*/*w*)	Size (nm)	PDI
0.5	301.4 ± 70.94	0.306 ± 0.081
1	195.7 ± 26.77	0.338 ± 0.093
1.5	138.7 ± 9.923	0.291 ± 0.069
2	115.1 ± 0.873	0.246 ± 0.008

**Table 2:** Size, PDI, ZP, and percent entrapment efficiency of SLNs loaded with different concentrations of nicotine (*n* = 3).

Nicotine (% *w*/*w*)	Size (nm)	PDI	ZP (mV)	Entrapment Efficiency
0.1	114.4 ± 2.33	0.213 ± 0.012	−48.2 ± 3.49	4.59 ± 18.81
0.2	136.0 ± 23.47	0.271 ± 0.061	−46.1 ± 7.56	11.51 ± 1.93
0.25	108.3 ± 2.77	0.201 ± 0.010	−29.6 ± 6.95	15.66 ± 15.38
0.5	112.3 ± 1.54	0.197 ± 0.008	−46.6 ± 4.89	11.15 ± 2.59
1.0	119.3 ± 2.70	0.151 ± 0.008	−52.4 ± 4.54	16.47 ± 5.64

**Table 3:** Formula for nicotine−stearic acid conjugate-loaded SLNs.

Ingredient	Quantity
Nicotine−stearic acid conjugate (equivalent to 50 mg of nicotine)	156 mg
Compritol 888 ATO	500 mg
Poloxamer 188	300 mg
Milli-Q water (quantity sufficient)	20 mL

**Table 4:** Effect of storage on particle size, PDI, and ZP of nicotine−stearic acid conjugate SLNs at 4 °C and RT.

	Storage Condition					
	4 °C	RT	4 °C	RT	4 °C	RT
Time (Days)	Particle Size (nm)		PDI		ZP (mV)	
0	113.5 ± 00.91	113.5 ± 0.91	0.211 ± 0.01	0.211 ± 0.01	−48.1 ± 5.75	−48.1 ± 5.75
30	128.3 ± 10.40	116.0 ± 3.36	0.291 ± 0.04	0.230 ± 0.011	−43.1 ± 4.12	−51.3 ± 6.72

## Data Availability

The data presented in the manuscript are available on request from the corresponding author.
